# Fairy: fast approximate coverage for multi-sample metagenomic binning

**DOI:** 10.1186/s40168-024-01861-6

**Published:** 2024-08-14

**Authors:** Jim Shaw, Yun William Yu

**Affiliations:** 1 Department of Mathematics, University of Toronto, Toronto, Canada; 2 Computational Biology Department, Carnegie Mellon University, Pittsburgh, USA

**Keywords:** Metagenomic binning, Coverage calculation, K-mers, Alignment-free

## Abstract

**Background:**

Metagenomic binning, the clustering of assembled contigs that belong to the same genome, is a crucial step for recovering metagenome-assembled genomes (MAGs). Contigs are linked by exploiting consistent signatures along a genome, such as read coverage patterns. Using coverage from multiple samples leads to higher-quality MAGs; however, standard pipelines require all-to-all read alignments for multiple samples to compute coverage, becoming a key computational bottleneck.

**Results:**

We present fairy (https://github.com/bluenote-1577/fairy), an *approximate* coverage calculation method for metagenomic binning. Fairy is a fast k-mer-based alignment-free method. For multi-sample binning, fairy can be $> 250 \times$ faster than read alignment and accurate enough for binning. Fairy is compatible with several existing binners on host and non-host-associated datasets. Using MetaBAT2, fairy recovers $98.5\%$ of MAGs with $> 50\%$ completeness and $< 5\%$ contamination relative to alignment with BWA. Notably, multi-sample binning with fairy is *always* better than single-sample binning using BWA ($> 1.5\times$ more $>50\%$ complete MAGs on average) while still being faster. For a public sediment metagenome project, we demonstrate that multi-sample binning recovers higher quality Asgard archaea MAGs than single-sample binning and that fairy’s results are indistinguishable from read alignment.

**Conclusions:**

Fairy is a new tool for approximately and quickly calculating multi-sample coverage for binning, resolving a computational bottleneck for metagenomics.

Video Abstract

**Supplementary Information:**

The online version contains supplementary material available at 10.1186/s40168-024-01861-6.

## Background

Direct shotgun sequencing of microbiomes has allowed for the recovery of metagenome-assembled genomes (MAGs), unlocking unprecedented insights into the ecology of even unculturable organisms [1]. The computational process of generating MAGs first requires *assembling* the sequenced reads into contiguous sequences called *contigs*. After assembly, contigs are grouped into MAGs by *binning* through either automated algorithms [2–5] or manual curation [6].

Binning is done by leveraging consistent information across an *entire genome*. Such genomic signatures [7] include k-mer frequencies (e.g., tetranucleotide frequencies) and sequencing coverage, which are commonly used in binning algorithms. Combining coverage information from *multiple samples* is an effective way of increasing the resolving power of binning algorithms [8]. It has been shown that multi-sample coverage is vastly superior to single-sample coverage for binning and produces better MAGs that even quality-control software such as CheckM [9] may not be able to detect [10].

Coverage calculation is usually done by aligning reads back to contigs (e.g., using BWA [11] or BowTie2 [12]). For a project with *n* samples and *n* resulting assemblies, computing multi-sample coverage naively requires aligning each sample to each assembly, resulting in $n^2$ read-alignment runs. This quadratic scaling becomes prohibitive when the number of samples is large. Co-assembly, where all reads are assembled to give one set of contigs, is a potential solution, but co-assembly can be memory intensive and collapse similar strains [13]. Another method is split-binning [4], where all contigs from a set of assemblies are concatenated together and then aligned to. This is faster but still relatively time and memory-intensive. Thus, many large-scale studies still do single-sample binning [14].

If only coverage is needed, read alignment is computationally wasteful because the exact base alignments are not needed. Alignment-free methods are faster and provide an intriguing alternative. For example, pseudoalignment [15, 16] has been used for coverage calculation [17, 18]. Additionally, direct k-mer counts have also been used to separate strains in de Bruijn graphs [19] and applied to multi-sample coverage for binning [20].

### Our contributions

In this paper, we present a much faster, alignment-free method of computing multi-sample coverage for metagenomic binning. Our method, *fairy*, is built on top of our metagenomic profiler *sylph* [21], but fairy is specifically adapted for metagenomic binning of contigs.

Fairy’s algorithm is related to previously developed k-mer sketching algorithms [22, 23], but we are not aware of detailed investigations justifying their effectiveness for the specific task of MAG recovery. Thus, we investigate the effectiveness of fairy on a diverse set of environments and methods. We confirm that fairy recovers multi-sample bins of similar quality relative to alignment at a fraction of the runtime, justifying the usage of k-mer sketching techniques and paving the way for more computationally efficient pipelines.

## Methods

The key computational insight of fairy is that indexing metagenomic *reads* with a sparse set of k-mers is much more efficient than indexing *genomes* for coverage calculation. This is due to the redundancy of k-mers within the reads, allowing fairy to be 2–3 orders of magnitude faster than alignment. A variant of this technique was previously employed for compressively accelerated all-mapping, but that task still required base-level alignment, whereas fairy eschews that step [24].

### Repurposing a fast metagenomic profiler for approximate coverage computation

Fairy’s codebase was forked and independently developed from sylph, a metagenomic profiler we developed [21]. However, fairy is extended and repurposed for metagenomic binning. Fairy and sylph are algorithmically similar, but they differ in three ways:

1. *Default parameter choices*. We will make the differences explicit below.
2. *User interface*. This includes command line options, inputs, and output formats―fairy is a *coverage calculator* rather than a *metagenomic profiler*.
3. *A coverage variance computation step*. This is useful for some binners such as MetaBAT2 [2].

**Fig. 1:**
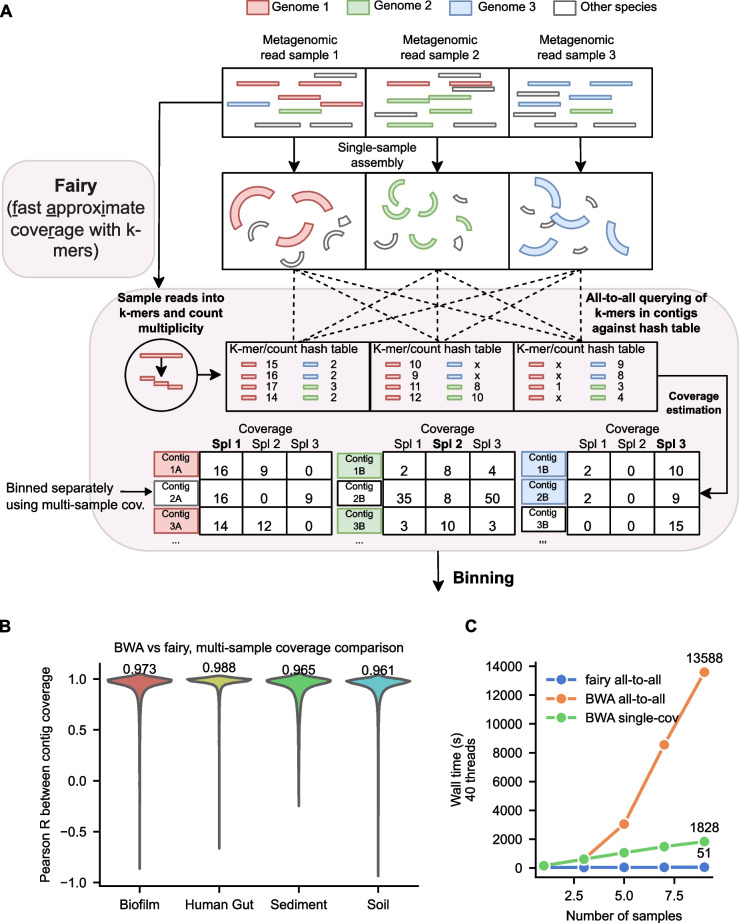
Fast approximate k-mer coverage estimates for multi-sample metagenomic binning. **A** Outline of fairy’s k-mer-based algorithm. Fairy’s processing steps are outlined in light red. Fairy indexes (or *sketches*) the reads into subsampled k-mer-to-count hash tables. K-mers from contigs are then queried against the hash tables to estimate coverage. Finally, fairy’s output is used for binning and is compatible with several binners (e.g., MetaBAT2, MaxBin2). Notice that white contigs (e.g., Contig 2A) have similar coverage to the colored contigs in the same sample, but additional samples help clarify that the white contigs should be binned separately. **B** Pearson *R* values between fairy and BWA’s multi-sample coverages for contigs in an arbitrary assembly from the dataset. Median values are shown above the plots. **C** Wall time with 40 threads for fairy vs BWA on 1, 3, 5, 7, and 9 gut samples in all-to-all mode (multi-sample) and single-sample mode

Fairy’s output is the same as the commonly used jgi_summarize_bam_contig_depths script from MetaBAT2 and can be toggled to be compatible with binners such as MaxBin2 and SemiBin2 as well. We recapitulate fairy’s key algorithmic steps below, also shown in Fig. 1A. We outline technical details pertaining to sylph’s algorithm in Additional file 1: Supplementary Methods.

#### Sparse sketching of k-mers

Fairy sparsely samples k-mers from reads and assemblies using the FracMinHash [25] method to sample approximately 1/50 k-mers (whereas sylph defaults to a 1/200 sampling rate). For each metagenomic sample, the k-mers in the reads and their associated multiplicities within the sample are stored in a hash table. The hash table indices (one for each sample) are written to disk and loaded as needed, whereas the assemblies’ k-mers are kept in memory.

#### Contig querying and containment

For an assembly, every contig’s sampled k-mers are queried against every metagenomic sample’s hash table. For a contig, fairy requires 8 k-mers at minimum to be contained in the sample (whereas sylph requires 50) to proceed with coverage calculation. In addition to the minimum k-mer threshold, fairy calculates a *containment ANI* in the same manner as sylph (Additional file 1: Supplementary Methods).

Containment ANI measures the average nucleotide identity of the contig to all sequences in the metagenome. Intuitively, the containment ANI is a continuous absence-presence measure for the contig in the reads. For example, if only one of a contig’s 1000 k-mers is present in the reads’ k-mers, it will have low containment ANI and is likely not present in the sample. If the containment ANI is $< 95\%$, fairy assumes the contig is not present in the sample at species-level, and thus assigns a coverage of 0.

We choose 95% as a presence-absence threshold by default, corresponding to species-level ANI thresholds used in practice [26]. Strain-level MAG binning from strain-resolved assemblies (e.g., with PacBio HiFi reads [27]) may require higher ANI thresholds, but higher thresholds would reduce sensitivity for multi-sample binning. In the “Results” section, we discuss some issues with the 95% threshold for PacBio HiFi MAG binning.

### Coverage calculation from k-mers

For a contig passing the containment ANI threshold, the coverage is estimated in three different ways. Let *M* be the median k-mer multiplicity of the contig’s k-mers within the sample. We calculate the coverage exactly as is done with sylph’s “effective coverage” estimator [21], briefly restated below (see Additional file 1: Supplementary Methods for more information). Fairy’s output coverage is:

- If $M \le 3$: a statistical estimator using Poisson coverage assumptions.
- If $4 \le M \le 15$: a robust mean of k-mer counts, trimming off large k-mer counts.
- If $M > 15$: median of the k-mer counts.

#### Coverage variance calculation

The variance is calculated as the sample variance of the contig’s non-zero k-mer multiplicities in the sample with the 10 and 90 percentile k-mer multiplicities trimmed to remove long-tailed k-mer coverage outliers (e.g., due to mobile elements).

### Why fairy is fast for multi-sample computation

Fairy’s speed comes from the fact that each sample’s reads are only processed once. Processing reads into hash tables turns out to be far more computationally costly than querying a contig’s k-mers against the hash table. Thus, for *n* samples, fairy only requires *n* costly read-processing steps and $n^2$ fast query procedures. This makes fairy scale well for multi-sample binning compared to read alignment.

### Benchmarking procedure

We benchmark on real datasets with multiple samples [28–34] and subsetted the samples when the number was $> 24$, the size of the largest dataset, to avoid extremely long computations; exact accessions are available in Additional file 3: Supplementary Table 2. We opted to benchmark on real data using CheckM2 as opposed to synthetic data to show that fairy has good performance on realistic datasets. It has been found that CheckM’s relative performance is strongly correlated with ground-truth performance on simulated data [35]. In particular, we primarily care about relative results rather than true contamination/completeness.

#### Assembly and MAG generation

For generating assemblies and MAGs using read alignment for binning, our procedure was as follows:

1. *Short-read metagenomes*: we generated assemblies using ATLAS [36] v2.18.1 with default settings. We mapped short-reads using BWA and used CoverM [37] with the -m metabat option, which is identical to using the jgi_summarize_bam_contig_depths script from MetaBAT2 with default settings.
2. *Nanopore long-read metagenomes*: we generated assemblies using metaFlye [38] with default settings. We mapped reads using minimap2 [39] and used the jgi_summarize_bam_contig_depths script with minimum mapping quality 5 and alignment identity $80\%$ (as opposed to default 97%) due to lower accuracy of nanopore reads.
3. *PacBio HiFi metagenomes*: we used MetaMDBG [40] for assembly and minimap2 with jgi_summarize_bam_contig_depths (default settings) for coverage calculation.

For all above data types, fairy v0.5.1 was then used with default parameters on the resulting assemblies and reads to generate another set of coverage profiles. Fairy’s coverage profiles were used to generate another set of MAGs that we compared to the MAGs generated from alignment-based coverages.

#### Binning and evaluation

For short-read assemblies, binning was conducted with MetaBAT2 v2.15 [2], MaxBin2 v2.2.7 [3], MetaBinner v1.4.4 [5], VAMB v3.0.9 [4], and SemiBin v2.1 [41] (with the -a option). Notably, we used an older version of VAMB because the newer version required BAM inputs, which fairy can not generate. For long-read assemblies, we only used MetaBAT2, as it seemed to be more commonly used for existing long-read pipelines [27, 33, 40]. All binners were run with standard settings, except using a minimum contig length of 1500 when possible to emulate existing pipelines [10]. Some binners can only be run with a BAM file as opposed to a custom coverage profile, so we could not benchmark them [42]. Finally, we use CheckM2 [9] to evaluate the contamination and completeness of the resulting bins.

## Results

### Fairy is concordant with read-alignment coverage while being $> 100$ times faster

For four short-read datasets (Fig. 1B), we took one arbitrary assembly and examined all present contigs. For *n* samples, each contig has *n* coverage values. We calculated the Pearson correlation between the *n* coverages that fairy output and BWA’s *n* coverages. The median Pearson R value was $> 0.96$ for all datasets, indicating good concordance. Sediment and soil metagenomes are often more complex (i.e., more low-abundance organisms and overall diversity) than gut metagenomes, likely explaining the lower concordance between BWA and fairy on these datasets (0.988 for gut vs 0.965 for sediment and 0.961 for soil).

Fairy is more than $250 \times$ faster than BWA for multi-sample coverage (Fig. 1C) when using just 9 of the short-read human gut metagenomes. While fairy technically requires a quadratic number of coverage calculations, much of the processing is in the linear-time indexing step. For read-alignment, the quadratic time cost of all-to-all alignment is clear for even a small number of samples.

#### Multi-sample coverage calculation quickly becomes a bottleneck, but not for fairy

For the soil dataset (10 samples) with 40 cores, assembly with SPAdes took approximately 15 h, while read alignment took more than 40 h. Theoretically, if we used 100 samples instead of 10, assembly would take $\approx$ $15 \times 10 = 150$ h and finish within a week, whereas all-to-all alignment would take $40 \times 10^2 = 4000$ h (around 167 days), which is not feasible. On this dataset, fairy took 9 minutes for indexing and 7 minutes for querying; most of the querying time was spent on disk I/O, and we did not even use an SSD. In the worst case, 100 samples would still take less than a day for fairy.

For memory, fairy scales with the size and complexity of the metagenome. Processing the 10 aforementioned soil samples in parallel took 40 GB of RAM (4 GB per sample), which is still relatively small compared to assembly, and if memory is a constraint, fewer samples can be processed in parallel.

### Multi-sample binning with fairy is better than single-sample binning with alignment

**Fig. 2:**
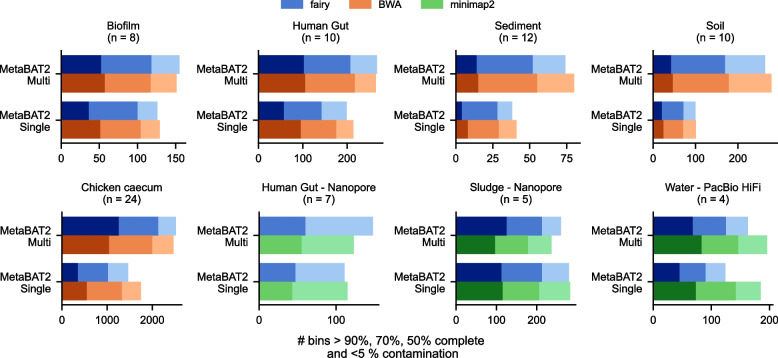
Binning results over multiple datasets for MetaBAT2. Fairy’s concordance with BWA and minimap2 for multi-sample and single-sample coverage binning using MetaBAT2. Darker shades indicate higher completeness (90%, 70%, and 50% completeness thresholds with < 5% contamination). minimap2 was used with long-read datasets (PacBio HiFi or nanopore), whereas BWA was used with short-read datasets. Sample accessions are available in Additional file 3: Supplementary Table 2

We compared fairy’s binning results with BWA and minimap2 using MetaBAT2 as the binner (Fig. 2). For every short-read dataset, fairy’s multi-sample binning outperforms single-sample binning, even with read alignment. This is especially prominent for complex sediment or soil metagenomes. We also note that single-sample binning contains contamination which may not be detectable by CheckM [10]. The long-read sludge metagenome is an exception, with minimap2 performing worse for multi-sample binning. We speculate that this is due to parameters (i.e., for coverage and binning) not being tuned as carefully for long reads. Compared to short-read binning, optimal parameter choices have not been explored as much for long-read binning―we do not claim our choice of alignment-based coverage parameters is optimal.

### Fairy recovers comparable quality bins to alignment for multi-sample short-read metagenomes

**Fig. 3:**
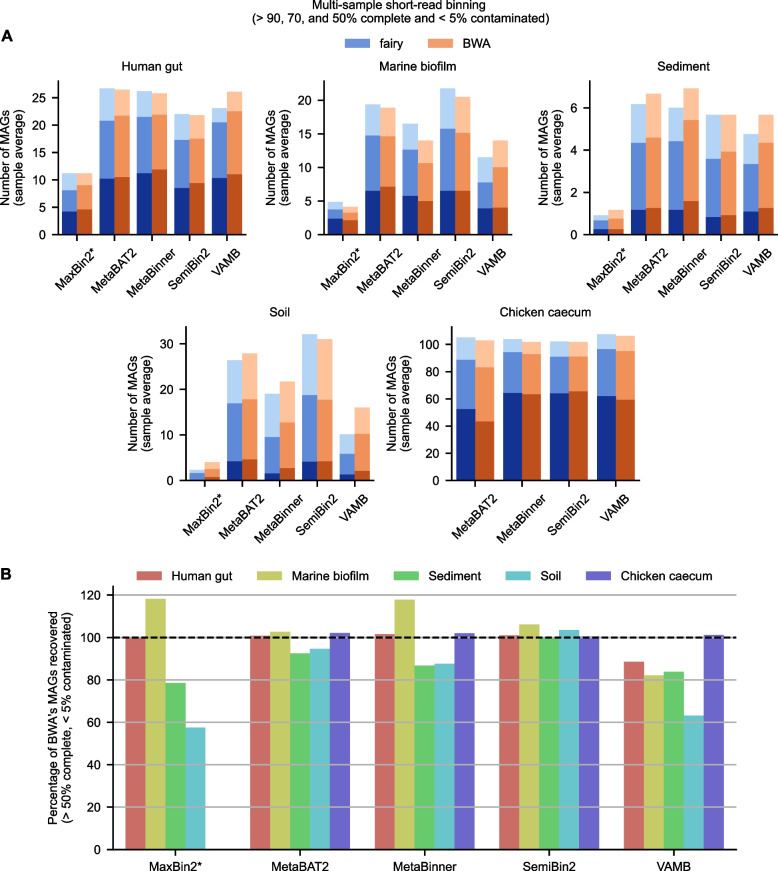
Short-read, multi-sample binning comparison across binning algorithms. **A** Number of bins recovered on average per sample with $> 50, 70,$ or $90\%$ completeness and $< 5\%$ contamination. Darker indicates higher completeness. *MaxBin2 did not complete on the chicken caecum dataset due to a software error. **B** Percentage of bins in (**A**) with $> 50\%$ completeness and $< 5\%$ contamination obtained with fairy relative to BWA over four different binners. This was calculated as $100 \times (\#\ \text{of fairy bins}) / (\#\ \text{of BWA bins})$. Higher than 100% indicates superior performance to BWA. Complete results are available in Additional file 2: Supplementary Table 1

Next, we analyzed fairy versus BWA across different binning algorithms (Fig. 3 and Additional file 1: Supplementary Figure 1). We focus on *multi-sample, short-read* binning. Overall, no binning method or coverage method was consistently the best (Fig. 3A). The mean percentage of recovered bins (> 50% complete; < 5% contaminated) as a percentage of BWA’s # of bins is 88.6%, 98.5%, 99.1%, 83.8%, and 102.2% for MaxBin2, MetaBAT2, MetaBinner, VAMB, and SemiBin2 respectively (Fig. 3B).

MaxBin2 produced very few bins on the soil and sediment datasets, so fairy’s large relative recovery dropoff represents a small absolute dropoff. We also note that for VAMB, our single-sample assembly with multi-sample coverage approach differs from the “multisplit” approach that VAMB suggests using, perhaps impacting performance. MaxBin2 failed to complete for the chicken caecum dataset where fairy outperformed BWA on all other binners (102%, 102%, 101%, and 100% recovered compared to BWA for MetaBAT2, MetaBinner, VAMB, and SemiBin2 respectively).

On our host-associated datasets (human gut and chicken caecum), fairy produces more $> 50\%$ complete bins than BWA for all binners except VAMB. Fairy particularly excelled for high-quality bins ($> 90\%$ complete and $<5\%$ contaminated) on the caecum dataset; fairy recovered 120% of BWA’s bins on the caecum dataset using MetaBAT2, a 20% increase in recovery. In Additional file 1: Supplementary Figure 2, we show an example where MetaBAT2 recovered a 98% complete bin with fairy, but the bin was split into two using BWA’s coverages.

#### Fairy’s bins are genomically similar to bins recovered by alignment

**Fig. 4:**
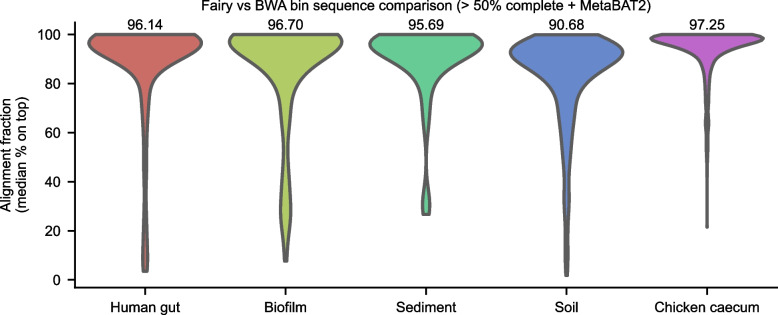
Alignment fraction of fairy’s bins versus BWA’s bins (multi-sample, short-read bins with MetaBAT2). For each sample, fairy’s bins with $> 50\%$ completeness were compared to all of BWA’s bins using skani [43]. For each bin, the highest alignment fraction match with $> 99\%$ ANI was taken. Median alignment fraction values are shown above each violin plot

We also verified that fairy’s short-read, multi-sample bins have similar genomic content to BWA’s bins, and not just similar completeness and contamination statistics (Fig. 4). We compared MetaBAT2 + fairy’s bins against MetaBAT2 + BWA’s bins across the same sample using skani [43]. For each of MetaBAT2 + fairy’s bins, we considered the highest aligned fraction match with $> 99\%$ ANI as the best hit. Over all of fairy’s $> 50\%$ complete bins, we found that the median aligned fraction against the best hit BWA bin was $> 95\%$ on all datasets except soil, which still had $> 90\%$ median aligned fraction. Thus more than half of fairy’s bins shared 90% of the same genomic content as one of BWA’s bins.

### When to use fairy versus alignment

Our results suggest that fairy performs well for multi-sample binning and especially for host-associated metagenomes. We outline two situations below where our results suggest that fairy can not yet replace read alignment.

#### Caveat 1: fairy is not as good as alignment for single-sample binning

For *single-sample* coverage with short reads, the performance of fairy is noticeably worse than BWA (Fig. 2). Using MetaBAT2, fairy recovers only 60% of BWA’s high-quality ($> 90\%$ completeness and $< 5\%$ contamination) human gut bins for single-sample binning, but 97% of BWA’s high-quality bins for multi-sample binning. We do not recommend fairy for single-sample binning―single-sample coverage calculation is not a bottleneck anyway.

#### Caveat 2: fairy is usable with nanopore long-reads, but not high-fidelity reads

For nanopore long-reads, our MetaBAT2 results show that fairy is competitive with minimap2’s alignment coverage (Fig. 2). However, fairy’s results are markedly worse than minimap2 when using PacBio HiFi reads. We found that this is because HiFi assemblers assemble similar strains instead of collapsing them, and our k-mer-based approach is not suitable for calculating coverage for extremely similar strains.

It may however be possible to tune fairy’s ANI thresholds for strain-level HiFi binning. Although PacBio HiFi metagenomic samples are still uncommon relative to short reads, we believe that strain-level binning is an interesting avenue to explore for future work. Nevertheless, we do not recommend fairy in its current version for PacBio HiFi MAG recovery.

### Case study: fairy recovers comparable Asgard archaea genomes in sediment

**Fig. 5:**
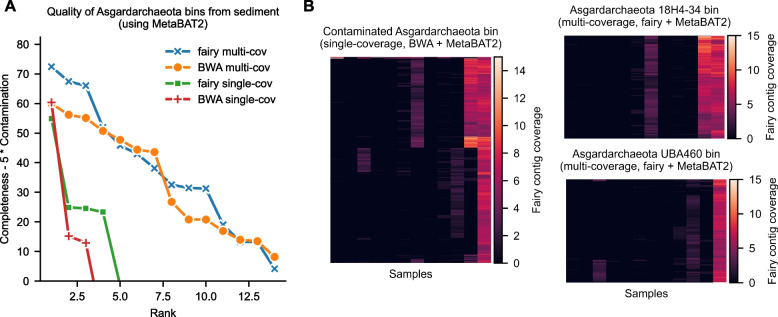
Investigating Asgard archaea from sediment metagenomes using MetaBAT2 with fairy and BWA. **A** Multi-sample binning, whether with fairy or BWA, recovers much higher quality Asgard archaea bins. **B** Coverage-contig heatmaps for specific bins. Left: single-sample binning produces a contaminated bin with two Asgard archaea (BWA’s bin pictured). Coverage patterns (fairy’s coverage) show clear contamination. Right: multi-sample binning disentangle the two genomes and correctly generates two bins (fairy’s bins pictured)

The study of Asgard archaea (or Asgardarchaeota) has served an important role in the study of eukaryogenesis [44]. Asgard archaea genomes were first recovered through metagenomics [45], and metagenomics continues to be important in their study [30]. In Fig. 5, we investigate the quality of Asgard archaea MAGs recovered from the sediment dataset (also used in Figs. 2 and 3). Sediment metagenomes are known to be complex [46], so this presents an interesting challenge for fairy.

For MetaBAT2 bins from the sediment metagenomes, we compared single-sample versus multi-sample binning and found that multi-sample binning produces much higher quality Asgard bins (Fig. 5A). Fairy is even slightly better than BWA when ranking the bins under the Completeness $- 5 \times$ Contamination metric.

We visualized an explicit example of where single-sample coverage binning failed in Fig. 5B. In the sample DRR310882, both fairy and BWA (with MetaBAT2) produced a contaminated bin with two Asgard archaea. BWA’s bin (visualized in Fig. 5B, left) had a 55% contamination estimate. The two erroneously binned Asgard genomes have similar coverage in this sample and could not be separated with k-mer frequencies. However, multi-sample coverage information disentangles the two genomes, and both fairy and BWA do so successfully. Fairy’s resulting multi-sample bins (Fig. 5B, right), with genus assignments 18H4-34 and UBA460 using GTDB-Tk [47], had only 2.66% and 4.25% contamination respectively.

## Discussion

### Fairy’s role in MAG recovery pipelines

Fairy is orders of magnitude faster than read alignment and solves a key computational bottleneck. The speed increase is not controversial. In some cases, fairy may give better results (e.g., the biofilm and chicken caecum datasets in Fig. 2), but there may be a slight sensitivity loss compared to read alignment on complex metagenomes such as sediment or soil. However, we show that when all-to-all alignment is not feasible, multi-sample binning with fairy should always be preferred over single-sample read alignment, thus filling an important niche for large-sample projects.

Furthermore, we envision fairy as complementary to all-to-all read alignment. Users can first use fairy to obtain a set of good-quality multi-sample bins and immediately analyze their data while they wait days/weeks for their all-to-all read alignments finish.

### For default parameters, fairy’s accuracy is encouraging

Binning accuracy depends on both the binner *and* the coverage calculation method. We used standard pipelines for obtaining read-alignment coverage and default parameters for all binners so that our results represent a realistic situation where fairy is used instead of, say, BWA. We have seen that fairy’s coverages have specific patterns not seen in read-alignment coverages (e.g., 0-coverage dropouts due to ANI thresholds; see Additional file 1: Supplementary Figure 2). It would be interesting to understand how binning parameters work in conjunction with coverages obtained by k-mer sketching techniques like fairy, and if alternative parameterizations could lead to better results.

## Conclusion

In this paper, we provide a new k-mer-based coverage calculation method for metagenomic binning called fairy. Fairy is magnitudes faster than read alignment and enables multi-sample binning on much larger datasets than before. In particular, we show that recovered bins are of competitive completeness and contamination to read-mapping approaches and sometimes even better.

Interesting future avenues to explore include investigating other approaches to calculate coverage for binning as well as understanding theoretically what coverage characteristics lead to good MAG recovery. We do not yet understand the exact tradeoffs, pitfalls, and issues for how coverage calculation affects binning. While we expect an inevitable speed-accuracy tradeoff across different methods, a deeper understanding would guide practitioners, allowing optimal MAG recovery pipeline design for their needs.

### Supplementary Information

Additional file 1: Supplementary Methods. Supplementary Figures.

Additional file 2: Supplementary Table 1.

Additional file 3: Supplementary Table 2.

## Data Availability

No new datasets were generated or analyzed during the current study. All analyzed samples are publicly available and accessions can be found in Additional file 3: Supplementary Table 2. Scripts for reproducing figures can be found at https://github.com/bluenote-1577/fairy-test/.
