# Supplementary material for: Fairy: fast approximate coverage for multi-sample metagenomic binning
Source: Microbiome. 2024 Aug 14;12:151. doi: 10.1186/s40168-024-01861-6 (PMC11323348; doi:10.1186/s40168-024-01861-6)
Supplement: Supplementary file 2 — Additional file 1: Supplementary Methods. Supplementary Figures. [file 40168_2024_1861_MOESM1_ESM.pdf]

A Supplementary Methods

A.1 FracMinHash for k-mer sampling

FracMinHash [1] is a method for systematically selecting a subset of k-mers from a larger set of k-mers (i.e. subsampling). All k-mers used in fairy are obtained after selection by FracMinHash. We describe FracMinHash below.

Let $h : \Sigma^k \to \{0, ..., 2^{64} - 1\}$ be a hash function from k-mers to 64-bit integers. Let c be a positive number. Given a set of k-mers X, we obtain a subset

$$FMH_c(X) = \{x \in X : h(x) < \frac{2^{64} - 1}{c}\}.$$

Intuitively, FracMinHash returns a set of k-mers approximately c times smaller than the original set, assuming a reasonably uniform hash function. The key reason we use FracMinHash is that k-mers are *consistently* subsampled across different sequences – given a fixed hash function, if a k-mer is sampled on a contig, the same k-mer will be sampled on the read (because its hash value is the same).

In practice, fairy uses minimap2's [2] hash function. Fairy uses $c = 50$ by default and $k = 31$.

A.2 Containment ANI

Let A be a contig's k-mers after applying FracMinHash. Let B be a metagenomic sample's (i.e. a set of reads) collection of k-mers after applying FracMinHash (over *all* reads). The naive containment ANI is defined as

$$ANI_{naive} = \left(\frac{|A \cap B|}{|A|}\right)^{1/k}.$$

The containment ANI generalizes the standard ANI for genome-to-genome comparisons [3] under a simple statistical model. The naive containment ANI is not accurate when the coverage of the contig is low within the sample [4]. To deal with this, sylph [5] introduced a statistical procedure to estimate containment ANI accurately under low coverage.

Briefly, let N_a be the number of k-mers in A (the contig) with multiplicity/count a in B (the sample). Under stochastic sequencing assumptions, we can estimate the *effective coverage* as $\lambda = (a+1)\frac{N_{a+1}}{N_a}$ [5, 6] for any $a > 0$; a is chosen to correspond to the largest value of N_a. Then, fairy's containment ANI can be estimated as

$$ANI = \left(\frac{|A \cap B|}{|A|(1 - e^{-\lambda})}\right)^{1/k}.$$

In practice, fairy only estimates λ when the median k-mer coverage in the sample is ≤ 3, otherwise we use the naive formula. Additional implementation details are given in the Methods section of sylph's manuscript [5].

A.3 Coverage calculation

Given a set of multiplicities for the k-mers in A (the contig) in the metagenomic sample, we calculate coverage as follows. Let M be the median k-mer multiplicity.

- If $M \leq 3$: fairy outputs the λ estimate (discussed previously).
- If $4 \leq M \leq 15$: fairy uses a robust mean as follows. Let $Z \sim Pois(M)$ be a Poisson random variable with mean M, the median k-mer multiplicity. We discard all k-mer multiplicities α with $P(Z > \alpha) < 10^{-10}$, and then output a robust mean. This is done to discard long-tailed outliers due to repetitive and shared k-mers.
- If $M > 15$, fairy outputs the median k-mer multiplicity M.

The intuition for the above procedure is that for small M, a statistical method is crucial because means and medians do not give enough resolution. For moderate M, means give better resolution than medians but require thresholding to ensure robustness to outliers.

B Supplementary Figures

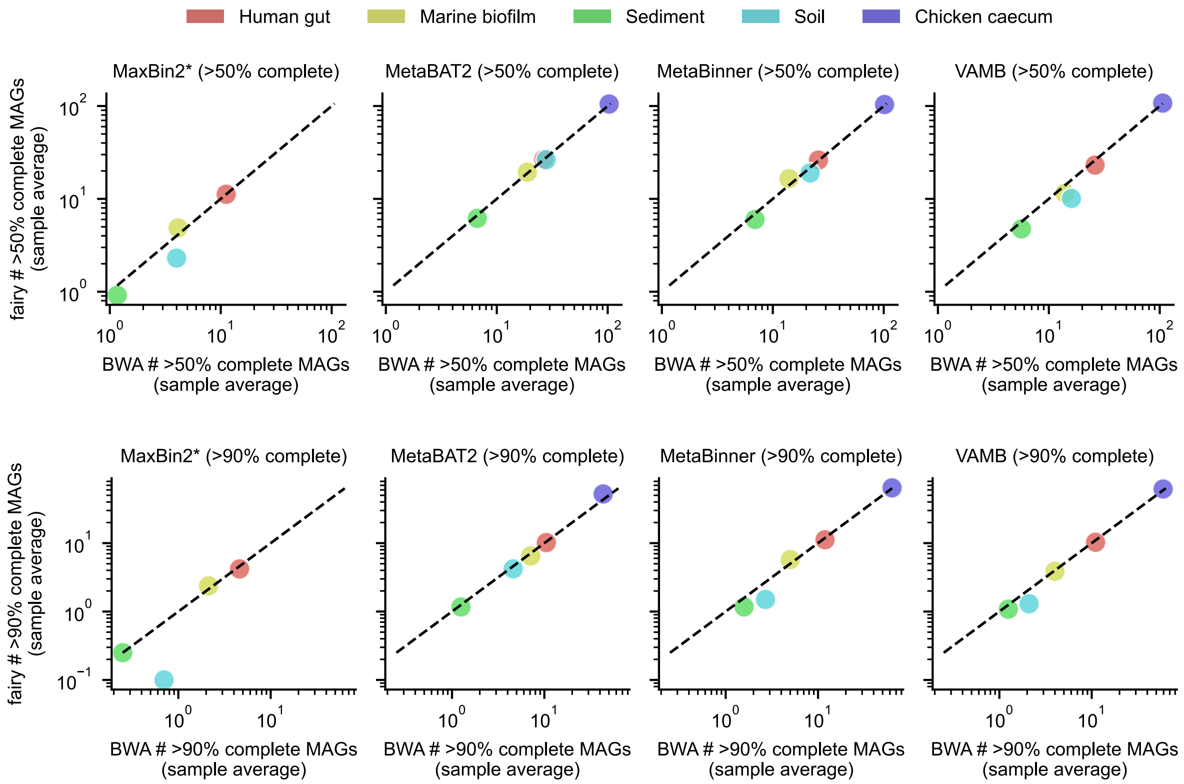

Fig. 1 Log-log plot of short-read, multi-sample bins recovered stratified by binning method. Top: $> 50\%$ complete and $< 5\%$ contaminated bins. Bottom: $> 90\%$ complete and $< 5\%$ contaminated bins.

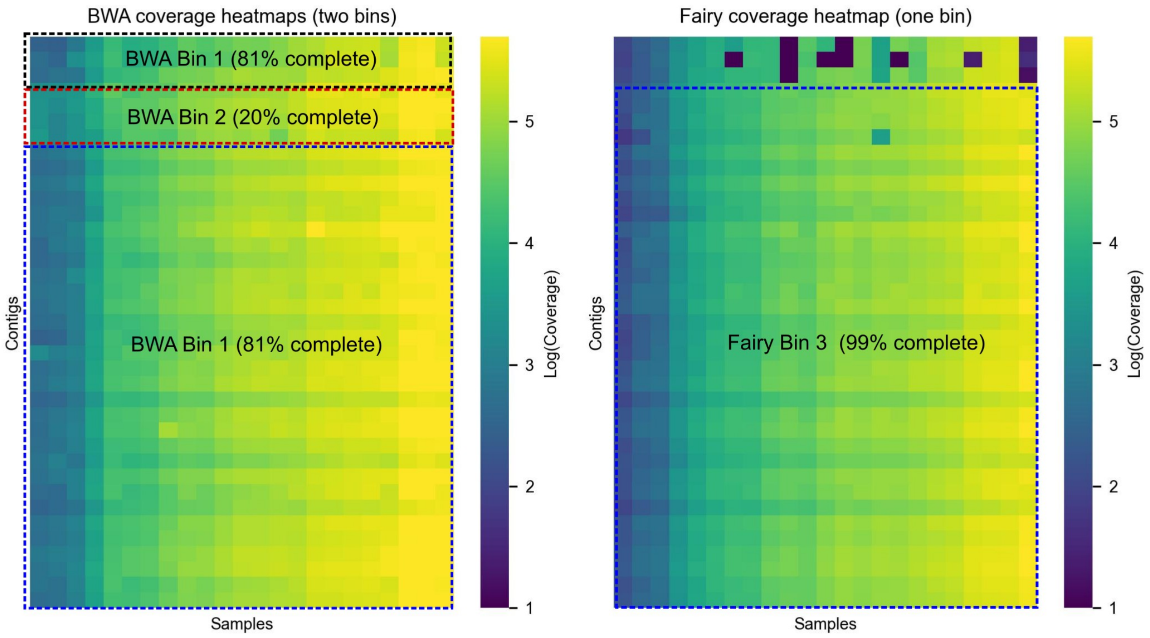

Fig. 2 Coverage patterns for fairy vs BWA on a high-quality bin. A high-quality bin on the chicken caecum dataset for fairy (right) was split into two bins for BWA (left) by MetaBAT2. The contigs on the top of fairy's heatmap were unique to BWA's results and not found in fairy's 99% complete bin. Coverage gaps (right, top) were due to the contigs not passing fairy's ANI thresholds. For the first column (i.e. sample), each of BWA Bin 2's contig coverages was larger than *all* of BWA Bin 1's coverages. However, this was not the case when analyzing BWA Bin 2's contigs using *fairy's* coverage.

References

[1] Irber, L., Brooks, P.T., Reiter, T., Pierce-Ward, N.T., Hera, M.R., Koslicki, D., Brown, C.T.: Lightweight compositional analysis of metagenomes with FracMinHash and minimum metagenome covers, 2022–0111475838 (2022) https://doi.org/10.1101/2022.01.11.475838 [New Results]. Chap. New Results

[2] Li, H.: Minimap2: Pairwise alignment for nucleotide sequences. Bioinformatics **34**(18), 3094–3100 (2018) https://doi.org/10.1093/bioinformatics/bty191

[3] Richter, M., Rosselló-Móra, R.: Shifting the genomic gold standard for the prokaryotic species definition. Proceedings of the National Academy of Sciences **106**(45), 19126–19131 (2009) https://doi.org/10.1073/pnas.0906412106

[4] Ondov, B.D., Treangen, T.J., Melsted, P., Mallonee, A.B., Bergman, N.H., Koren, S., Phillippy, A.M.: Mash: Fast genome and metagenome distance estimation using MinHash. Genome Biology **17**(1), 132 (2016) https://doi.org/10.1186/s13059-016-0997-x

[5] Shaw, J., Yu, Y.W.: Metagenome profiling and containment estimation through abundance-corrected k-mer sketching with sylph. bioRxiv, 2023–1120567879 (2023) https://doi.org/10.1101/2023.11.20.567879

[6] Sarmashghi, S., Bohmann, K., P. Gilbert, M.T., Bafna, V., Mirarab, S.: Skmer: Assembly-free and alignment-free sample identification using genome skims. Genome Biology **20**(1), 34 (2019) https://doi.org/10.1186/s13059-019-1632-4
